# High-Dose Ifosfamide Chemotherapy in a Series of Patients Affected by Myxoid Liposarcoma

**DOI:** 10.1155/2017/3739159

**Published:** 2017-08-30

**Authors:** Vittoria Colia, Elena Fumagalli, Salvatore Provenzano, Rossella Bertulli, Silvia Stacchiotti, Carlo Morosi, Paola Collini, Alessandro Gronchi, Paolo G. Casali, Roberta Sanfilippo

**Affiliations:** ^1^Medical Oncology Unit 2, Medical Oncology Department, Fondazione IRCCS Istituto Nazionale dei Tumori, Milan, Italy; ^2^Department of Radiology, Fondazione IRCCS Istituto Nazionale dei Tumori, Milan, Italy; ^3^Department of Diagnostic Pathology and Laboratory Medicine, Fondazione IRCCS Istituto Nazionale dei Tumori, Milan, Italy; ^4^Department of Surgery, Fondazione IRCCS Istituto Nazionale dei Tumori, Milan, Italy; ^5^Oncology and Haemato-Oncology Department, University of Milan, Milan, Italy

## Abstract

**Background:**

To report on the activity of high-dose prolonged-infusion ifosfamide (HDIFX) chemotherapy in a retrospective series of patients affected by myxoid liposarcoma treated at Fondazione IRCCS Istituto Nazionale dei Tumori in Milan, Italy.

**Patients and Methods:**

Patients with an advanced myxoid liposarcoma treated with HDIFX (14 g/sqm, i.v., prolonged infusion of 14 days every 28 days) as a single agent between May 2002 and April 2017 were retrospectively reviewed. All pathologic diagnoses were centrally reviewed and molecularly confirmed. Response was evaluated by RECIST, and survival functions were computed by the Kaplan-Meier method.

**Results:**

Eleven patients with advanced myxoid liposarcoma were treated with HDIFX (male/female = 9/2, median age 33 years, range 31–75). Among these, 1/11 received HDIFX in first line, 5/11 in second line, 3/11 in third line, and 2/11 in fourth line for a median course number of 3 (range 2–7). No RECIST objective responses were observed. Overall median progression-free survival was 1,9 months. Median overall survival was 37 months. At a median follow-up of 115 months, 1 patient is alive.

**Conclusions:**

In this series of patients affected by advanced myxoid liposarcoma, chemotherapy with HDIFX was essentially inactive.

## 1. Introduction

Liposarcoma is the most common histological subtype among soft tissue sarcomas. Specifically, myxoid/round cell liposarcoma is the second of the three liposarcoma subtypes, accounting for 30% of liposarcoma cases. Myxoid/round cell liposarcoma typically occurs around the fifth decade (range 26–81 years) at any site of the body, mainly in the lower extremities [1–4].

The diagnosis is supported by the detection of the characteristic chromosomal recurrent translocation, namely, t(12; 16)(q13; p11), which results in the aberrant fusion gene FUS-CHOP/DDIT3 [5]. According to the most recent WHO classification, myxoid liposarcomas can be morphologically divided into high and low grade [3]. Standard treatment for localized disease is surgery alone or in combination with radiotherapy, while medical treatment may be offered in selected cases and is often the main choice in the advanced setting [6–9]. Myxoid liposarcomas are known as being particularly sensitive to chemotherapy compared with other soft tissue sarcomas [8–10], in particular to anthracycline-based combinations and trabectedin. In the whole group of advanced soft tissue sarcomas, first-line combination of anthracycline and ifosfamide provides response rates around 20–40%. It increases to 65% in myxoid liposarcoma [8, 11, 12]. On the other side, the expected response rate of myxoid liposarcoma to trabectedin is 50% [13–18], with a mechanism of action which is apparently specific inasmuch as implying an effect of the drug on the binding of the fusion transcript to target genes [16–18]. Recently, another marine-derived drug, eribulin, was shown to be effective in metastatic adipocytic sarcomas, with a significant improvement in overall survival which led to its approval by FDA and EMA [19–21]. However, its activity in myxoid liposarcoma compared to pleomorphic and well-differentiated/dedifferentiated liposarcoma is still not defined. No other agents commonly used for treatment of advanced soft tissue sarcoma (e.g., gemcitabine, dacarbazine, and pazopanib) have been demonstrated to be active in myxoid liposarcoma [2].

High-dose prolonged-infusion ifosfamide (HDIFX) is known to be active in soft tissue sarcoma patients, even in those previously treated with standard-dose ifosfamide [2, 22–24]. A peculiar activity has been demonstrated in dedifferentiated liposarcoma, especially with a high malignancy grade component [10]. By contrast, no data are reported so far on the activity of high-dose ifosfamide in myxoid liposarcoma and no prospective neither retrospective studies focusing on this drug in the disease are available.

The aim of this retrospective analysis was to review all patients affected by advanced myxoid liposarcoma treated with high-dose prolonged-infusion ifosfamide at our Institution.

## 2. Materials and Methods

All patients affected by advanced myxoid liposarcoma, consecutively treated with HDIFX chemotherapy between May 2002 and April 2017 at Fondazione IRCCS Istituto Nazionale dei Tumori, Milan, Italy, were retrospectively identified. Pathological diagnosis was centrally reviewed by expert pathologists in all the cases, following the most recent updated criteria. Data regarding clinical and histopathological characteristics, staging, surgical and systemic treatment, and survival were collected. The clinical records were reviewed and collected in one institutional database and a descriptive analysis was performed.

High-dose ifosfamide was administered as a single agent, at the daily dose of 1 g/sqm (total dose of 14 g/sqm per cycle), as a 14-day continuous infusion with the same dose of MESNA every 4 weeks, through external portable infusion devices (elastomeric pumps) each providing a 7-day infusion, so that 2 pumps were used in each cycle. The infusion was stopped with no additional MESNA after day 14. No additional hydration was foreseen and only oral hydration with 1.5 L/day was recommended. When required, antiemetic prophylaxis was based on oral ondansetron, or dexamethasone, or metoclopramide. No granulocyte growth factors were administered. The treatment was continued until disease progression, serious adverse events, medical decision, or patient refusal. Data on chemotherapy tolerability and adverse effects were reviewed.

Treatment follow-up investigations included a whole-body computed tomography scan (CT), a magnetic resonance (MRI), or a CT of the primary site at baseline and then were generally repeated after the first two courses of chemotherapy and then every 2-3 months. Response Evaluation Criteria in Solid Tumours (RECIST) were used to assess response to chemotherapy [25].

This retrospective analysis was approved by the Institutional Ethics Committee.

### 2.1. Statistical Analysis

Progression-free survival (PFS) and overall survival (OS) were estimated with Kaplan-Meier method [26]. Progression-free survival death and progressive disease according to RECIST were considered as failures, and death due to any cause was failure for OS. Patients alive were censored at the time of the last contact.

## 3. Results

Within the study period, 11 patients affected by advanced myxoid liposarcoma treated with HDIFX were identified.

Patient characteristics are summarized in Table 1. Male patients were prevalent (male/female 9/2); median age at the time of the treatment was 33 (range 31–75). Median number of ifosfamide cycles per patient was 3 (range 2–7). Among these, 1/11 received HDIFX in first line, 5/11 in second line, 3/11 in third line, and 2/11 in fourth line.

All patients had previously been treated with anthracycline ± ifosfamide in adjuvant or advanced setting. Among these, 7/11 patients were treated with epirubicin plus ifosfamide in the adjuvant setting and 4/11 as first-line treatment for advanced disease with 1/4 complete response (CR), 2/4 for partial response (PR), and 1/4 for stable disease (SD). In the latter, median progression-free survival was 16.5 months (range 6–21), with the only patient with SD experiencing a PFS shorter than 1 year. Three/11 patients were pretreated with trabectedin as well, with 2 PR and 1 SD. All patients had metastatic disease when starting HDIFX, with a median of 2 sites involved (range: 1–3); among these, the lung was the most common involved site (8/11, 73%), followed by the abdominal cavity (7/11, 64%), the bone (1/11, 9%), and the soft tissues (1/11, 9%). Four patients (36%) had, in addition, a local relapse.

**Figure 1:**
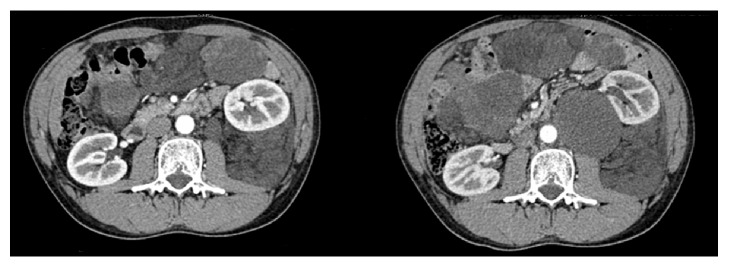
Multidetector computed tomography (MDCT) scan after contrast medium (arterial phase). Progression of abdominal metastasis from a myxoid liposarcoma after two cycles of high-dose prolonged-infusion ifosfamide.

All patients treated with HDIFX were evaluable for response. The best response was PD in 7/11 (36%) and SD in 4/11 (14%) with no PR or CR (Figure 1). Treatment was withdrawn because of progressive disease in all cases. No febrile neutropenia and renal failure were observed, as well as no toxic deaths or any other unexpected serious adverse events.

**Figure 2:**
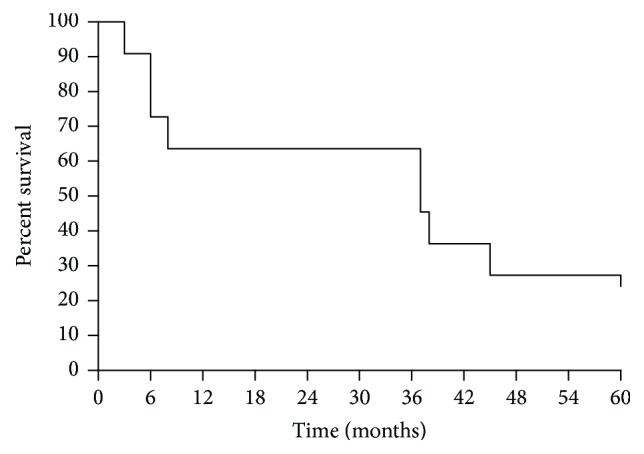
Overall survival (OS) of patients treated with high-dose prolonged-infusion ifosfamide chemotherapy (11 patients). Median OS was 37 months.

**Figure 3:**
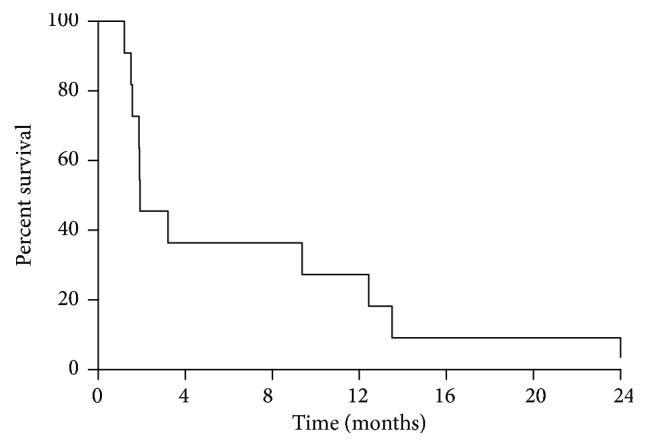
Overall progression-free survival (PFS) of patients treated with high-dose prolonged-infusion ifosfamide chemotherapy (11 patients). Median PFS was 1.9 months.

After a median follow-up of 115 months, OS was 37 months (Figure 2), with 10 patients dead and 1 patient alive at the time of this analysis. Median PFS was 1,9 months (Figure 3).

## 4. Discussion

In this small retrospective case-series of 11 patients with progressing advanced myxoid liposarcoma treated with chemotherapy on a 14-year span, no responses to continuous infusion high-dose ifosfamide were observed. Median PFS was 1,9 months, while median OS was 37 months.

Front-line ifosfamide combined with anthracyclines is clinically active in adult advanced soft tissue sarcomas, including liposarcomas [11, 12]. Following a histology-driven approach, high-dose ifosfamide as a single agent is often used as second-/further line treatment, even in patients previously treated with standard-dose ifosfamide.

HDIFX is known to be active in dedifferentiated liposarcoma and other histotypes [2, 10, 22–24, 27]. In our series, all patients had responded to anthracycline ± ifosfamide used as front-line treatment. These data are in line with studies pointing to a high sensitivity to cytotoxic chemotherapy of myxoid/round cell liposarcoma [8–10]. This is striking if compared to the apparent lack of activity of HDIFX in myxoid liposarcoma. Interestingly, there are no studies comparing the activity of anthracycline plus ifosfamide versus anthracycline alone in myxoid liposarcoma. On this basis, it is not known to which extent ifosfamide adds to anthracycline as a single agent in the first-line treatment of myxoid liposarcoma. The fact that in our series HDIFX did not show any antitumor activity raises doubts over its efficacy in myxoid liposarcomas. Clearly, all patients included in this series had been pretreated with doxorubicin plus ifosfamide, and we cannot rule out that this was an acquired resistance to the drug. However, the antitumor effect of ifosfamide as such may be better evaluated prospectively also in first line.

## 5. Conclusions

This was a retrospective analysis; the number of patients was limited and data refer to a 14-year span. However, it is difficult to extrapolate evidence pertaining to this subgroup of patients, let alone the lack of published retrospective and prospective studies on HDIFX selectively in myxoid liposarcoma. Thus, we believe that this series may stimulate collection of further cases. It is unfortunate that even if myxoid liposarcoma is considered chemosensitive among soft tissue sarcomas, only anthracyclines and trabectedin are active agents in this subtype at the moment. New medical treatments would be definitely needed.

## Figures and Tables

**Table 1:** Patients characteristics at chemotherapy when starting ifosfamide.

Patients characteristics	
*Age, years*	
Median	33
Range	31–75

*Histopathological diagnosis*	
Round cell myxoid liposarcoma (high grade)	7 (63%)
Classical myxoid liposarcoma (low grade)	4 (37%)

Metastatic disease at diagnosis	2 (18%)
Localized disease at diagnosis	9 (82%)

*Adjuvant chemotherapy*	
Y	8 (73%)
N	3 (27%)

*Adjuvant radiotherapy*	
Y	7 (64%)
N	4 (36%)

*Number of sites involved*	
Median	2
Range	1–3

*Local relapse*	4 (36%)

*Metastatic sites*	
Lung	8 (73%)
Abdominal cavity	7 (64%)
Bone	1 (9%)
Soft Tissue	1 (9%)

*Number of prior chemotherapy regimens*	
0	1 (10%)
1	5 (45%)
2	3 (27%)
3	2 (18%)

*Prior chemotherapy*	
Anthracycline ± Ifosfamide	11 (100%)
Trabectedin	3 (27%)
Pemetrexed	1 (10%)
Liposomal doxorubicin	1 (9%)
Unknown	2 (18%)

*High-dose ifosfamide infusion*	
Elastomeric pump	11 (100%)

*Number of ifosfamide courses*	
Median	3
Range	(2–7)
